# Cardiovascular magnetic resonance detects subclinical cardiac involvement in giant cell arteritis^☆^

**DOI:** 10.1016/j.jocmr.2026.102740

**Published:** 2026-04-27

**Authors:** Annemarie Proff, Simon M. Petzinna, Lena Kreis, Sophie-Marie Kirch, Taraneh Aziz-Safaie, Narine Mesropyan, Dmitrij Kravchenko, Anja Winklbauer, Tatjana Dell, Claus C. Pieper, Daniel Kuetting, Julian A. Luetkens, Valentin S. Schäfer, Alexander Isaak

**Affiliations:** a Department of Diagnostic and Interventional Radiology, University Hospital Bonn, Bonn, Germany; b Quantitative Imaging Lab Bonn (QILaB), University Hospital Bonn, Bonn, Germany; c Clinic of Internal Medicine III for Oncology, Haematology, Immune-Oncology and Rheumatology, University Hospital Bonn, Bonn, Germany

**Keywords:** Giant cell arteritis, Magnetic resonance imaging, Cardiovascular disease, Myocardial disease, Inflammation

## Abstract

**Background:**

To assess cardiac involvement in patients with newly diagnosed giant cell arteritis (GCA) using cardiovascular magnetic resonance (CMR).

**Methods:**

In this prospective single-center study, patients with newly diagnosed GCA underwent CMR at baseline and under therapy 6 months later. The imaging protocol enabled evaluation of cardiac function and volumes, edema, late gadolinium enhancement (LGE), and T1 and T2 mapping, including extracellular volume fraction (ECV). Healthy controls were included for comparison. Group comparisons were performed using t-tests, Mann–Whitney U, and chi-square tests. Paired t-tests assessed longitudinal changes.

**Results:**

A total of 45 GCA patients (mean age 73±9 years; 42.2% female) and 30 healthy controls were included. Active inflammatory cardiac disease was found in 3/45 (6.7%) of patients, comprising active pericarditis in 2/3 (66.7%) and active myocarditis in 1/3 (33.3%). LGE was observed in 12/45 (26.7%) of patients (5/12 [41.7%] with ischemic, 5/12 [41.7%] with non-ischemic, and 2/12 [16.7%] with pericardial pattern). Compared to controls, GCA patients had significantly elevated native T1 relaxation times (987±29 vs. 968±22 ms; p=0.003) and ECV values (27.3±3.8% vs. 25±2.1%; p=0.003). Patients with large-vessel involvement (24/45 [53.3%]) showed higher myocardial ECV values (28.7±4.2% vs. 25.8±2.5%, p=0.008). Follow-up CMR was performed in 35 patients; here, no significant changes were observed compared to baseline CMR (e.g., native T1: 984±25 vs. 980±20 ms, p=0.397).

**Conclusion:**

In patients with newly diagnosed GCA, CMR reveals subclinical cardiac involvement, including active inflammatory disease (myocarditis and pericarditis), post-ischemic scars, and signs of non-ischemic myocardial fibrosis.

## Graphical abstract

**Figure:**
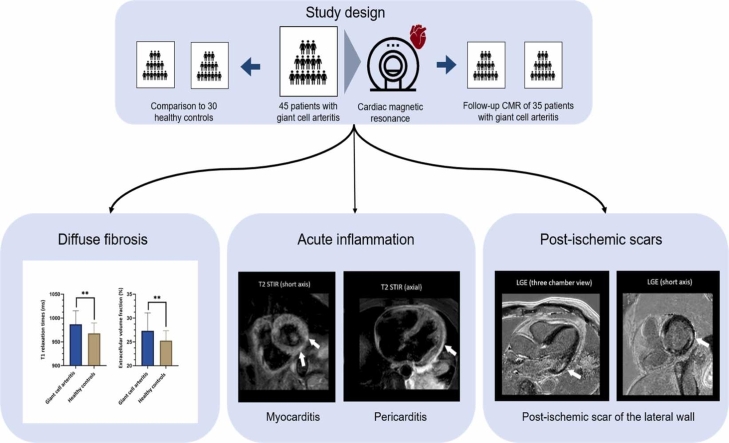

## 1 Introduction

Giant cell arteritis (GCA) is a systemic large-vessel vasculitis that primarily affects adults over the age of 50. It can involve multiple organs, including the cardiovascular system, and is associated with an increased risk of all-cause mortality [1]. While vascular complications such as aortitis, aneurysm formation, and dissection are well recognized, myocardial involvement in GCA remains poorly characterized. Case reports and case series suggest that myocardial injury may result from either direct inflammatory infiltration or secondary ischemic mechanisms [2]. Retrospective data further indicate that clinically significant myocarditis is rare in GCA, yet may present as a potentially life-threatening complication [3]. However, systematic, prospective imaging studies evaluating myocardial tissue abnormalities in patients with GCA are lacking.

As the non-invasive reference standard for evaluating cardiac function and myocardial tissue composition, cardiovascular magnetic resonance (CMR) enables comprehensive assessment through quantitative techniques such as T1 and T2 mapping and extracellular volume fraction (ECV) [4]. These parameters allow detection of diffuse myocardial abnormalities, including inflammation, edema, and fibrosis, and have been extensively validated in various cardiomyopathies and in systemic diseases 5, 6, 7, 8. For instance, subclinical myocardial tissue alterations have been described using quantitative CMR in other systemic autoimmune diseases, including rheumatoid arthritis and large-vessel vasculitis [9].

Given the limited evidence of cardiac involvement in GCA and the proven utility of quantitative CMR in detecting cardiac involvement in other systemic autoimmune diseases, we hypothesize that multiparametric CMR can reveal previously unrecognized cardiac involvement in newly diagnosed GCA.

## 2 Methods

This prospective single-center observational study was approved by the institutional ethics committee of the University of Bonn, Bonn, Germany. All patients gave written informed consent. The study is registered at ClinicalTrials.gov (identifier: NCT07031284).

### 2.1 Cohort characteristics

In this prospective clinical study, patients with newly diagnosed GCA were consecutively enrolled between December 2022 and March 2025. A healthy control group was included from historical data. Diagnosis of GCA was made by a board-certified rheumatologist also fulfilling the ACR-EULAR classification criteria [10]. Exclusion criteria included general contraindications for contrast-enhanced CMR (e.g., MRI-incompatible implants, known allergy to MRI contrast agents, severe renal impairment, and claustrophobia). All patients underwent a comprehensive ultrasound examination of the superficial temporal arteries and their branches, along with the facial, axillary, carotid, and vertebral arteries using a GE Logiq S10 system as previously described [11]. Intima-media thickness was measured in B-mode at the point of maximal thickness on the distal vessel wall and assessed using GCA-specific Outcome Measures in Rheumatology (OMERACT) 12, 13, 14. Large vessel involvement was defined by abnormal findings in axillary, subclavian, carotid arteries, or the aorta.

Patients were screened for cardiovascular risk factors and known underlying cardiac disease. Atherosclerosis was defined on the basis of documented medical history and chart review, including previously diagnosed coronary artery disease (CAD), with or without prior therapeutic procedures such as percutaneous coronary intervention (PCI) or coronary artery bypass grafting (CABG), peripheral artery disease, or atherosclerosis otherwise documented in the patients’ medical records. This information was supplemented by patient interviews during study inclusion.

The control group comprised retrospectively identified individuals without known cardiac disease and a normal CMR that was derived from an established control cohort previously used in a prior study [8] and additional controls from an ongoing unpublished study. All CMR scans were performed with a study-related indication between October, 2015 and December, 2019 on the same MRI system (Ingenia 1.5T, Philips Healthcare). More details are reported in Table 1.

**Table 1:** Clinical characteristics of the study population with giant cell arteritis (n=45) and the healthy control cohort (n=30)

**Parameter**	**Value**
Patients with Giant Cell Arteritis	
*Clinical parameters*	
Age (y)	73±9
Sex (female)	19/45 (42.2%)
Body mass index (kg/m²)	26.1±5.0
Body surface area (m²)	1.9±0.2
Heart rate (bpm)	68±13
Large vessel involvement	24/45 (53.3%)
OMERACT GCA ultrasonography score	1.2±0.3
*Laboratory parameters*	
C-reactive protein (mg/L)	23.4±28.7
Erythrocyte sedimentation rate (mm/h)	52.1±26.4
Leukocyte count ($10^{9}$/L)	9.6±3.2
Hematocrit (%)	38.4±5.1
*Clinical symptoms*	
Chest pain	1/45 (2.2%)
Headache	27/45 (60.0%)
Visual loss	26/45 (57.8%)
Visual impairment	31/45 (68.9%)
Temporal pain	20/45 (44.4%)
Tongue burning	21/45 (46.7%)
Jaw claudication	26/45 (57.8%)
Fatigue	36/45 (80.0%)
Weight loss	23/45 (51.1%)
Night sweat	8/45 (17.8%)
Shoulder/pelvic pain	16/45 (35.6%)
*Cardiovascular risk factors*	
Median of total cardiovascular risk factors	1 (IQR 0–2)
Obesity (Body Mass Index >30)	11/45 (24.4%)
Hypertension	21/45 (46.7%)
Diabetes	10/45 (22.2%)
Smoking	11/45 (24.4%)
Atherosclerosis	11/45 (24.4%)
*Final CMR diagnosis*	
Unremarkable/nonspecific	29/45 (64.4%)
Active inflammation	3/45 (6.7%)
− Myocarditis	1/3 (33.3%)
− Pericarditis	2/3 (66.7%)
Possible diffuse myocardial inflammation*	4/45 (8.9%)
Possible post-inflammatory fibrosis	4/45 (8.9%)
Post-ischemic scars	5/45 (11.1%)
Healthy controls	
*Clinical parameters*	
Age (y)	53±12
Sex (female)	14/30 (46.7%)
Body mass index (kg/m²)	25.6±5.4
Body surface area (m²)	1.96±0.22
Heart rate (bpm)	65±11

Data are numbers (%) of cases, means ± standard deviation, or medians (interquartile range). *OMERACT* Outcome Measures in Rheumatology, *GCA* giant cell arteritis, *CMR* cardiovascular magnetic resonance

* combined elevation of T1 and T2-relaxation-time in myocardial mapping

### 2.2 Cardiovascular magnetic resonance protocol

CMR was performed on a 1.5 Tesla MRI (Ingenia 1.5 Tesla, Philips Healthcare, Best, the Netherlands). Electrocardiogram-gated steady-state free precession cine sequences for analysis of functional and volumetric parameters were acquired in short-axis, two-chamber, and four-chamber views. For detection of visual myocardial edema, T2-weighted short-tau inversion-recovery sequences (STIR) were acquired (short-axis and transversal views). Modified gradient echo and spin echo sequences (GraSe) were used for myocardial T2 mapping. For T1 mapping, a modified Look-Locker inversion recovery was acquired before and 10 min after intravenous application of contrast agent as previously described 4, 6. T1 and T2 mapping sequences were acquired in end-diastolic short-axis view covering an apical, midventricular, and basal slice. Late gadolinium enhancement (LGE) sequences were acquired using phase-sensitive inversion recovery technique in standardized long-axis and short-axis view. A bolus of 0.2 mmol/kg body weight of gadoterate meglumine (Clariscan, GE Healthcare, Munich, Germany) was used for contrast enhancement.

### 2.3 Image analysis

Two cardiovascular radiologists (A.P., A.I.) performed CMR analysis in consensus agreement blinded to the clinical data using dedicated software (Medis Suite 4.0.70.4, Medis Medical Imaging Systems B.V., Leiden, the Netherlands and Philips IntelliSpace Portal v.10.1, Philips Healthcare). Left and right ventricular function and volumes were assessed on short-axis cine images. Functional parameters were indexed to body surface area. LGE was evaluated visually (presence of myocardial or pericardial enhancement) with regard to location and distribution pattern. Ischemic LGE was defined as subendocardial or transmural enhancement in a coronary artery territory, whereas non-ischemic LGE included mid-wall, subepicardial, or diffuse enhancement. Myocardial edema was assessed visually on T2-STIR images as regional areas of increased signal intensity compared to adjacent normal myocardium by consensus agreement of the two readers. Semiquantitative T2 signal intensity ratio was determined according to established methods [6]. Myocardial global native T1 and T2 relaxation times were derived according to standardized segmentation and myocardial delineation (American Heart Association (AHA) 16-segment model). Global extracellular volume fraction (ECV) values were calculated using pre- and post-contrast T1 mapping as recommended [4]. T1 and T2 relaxation times were defined as elevated based on local reference values for inflammatory myocardial disease (T1 ≥ 1000 ms, T2 ≥ 55.9 ms) [15].

Left ventricular strain analyses were performed using the QStrain module (Medis Suite MR, version 4.0, Medis Medical Imaging Systems B.V.). Endocardial and epicardial LV contours were automatically delineated and propagated throughout the cardiac cycle, with manual adjustment if tracking was inadequate upon visual inspection. Strain parameters included global longitudinal strain (GLS) and global circumferential strain (GCS).

### 2.4 Statistical analysis

Statistical analysis was performed using SPSS (version 20.0.0.0 (241); IBM Corp., Armonk, New York) and Prism (version 10.4.1 (627); GraphPad Software, San Diego, California). Continuous variables are presented as mean ± standard deviation (SD) or as median with interquartile range (IQR), as appropriate. Categorical variables are reported as absolute numbers and percentages. Group comparisons were conducted using the independent samples t-test for normally distributed data, and the Mann–Whitney U test for non-normal distributions. The Chi-square test, Fisher´s Exact test, and McNemar test were used to compare categorical variables. Paired t-test was conducted to assess within-group differences. Correlations between myocardial tissue parameters (T1, T2, ECV), amount of cardiovascular risk factors and inflammatory markers were examined using Pearson’s correlation coefficient. Results are reported as odds ratios with 95% confidence intervals. A two-tailed p<0.05 was considered statistically significant.

## 3 Results

### 3.1 General results

A total of 45 patients with newly diagnosed GCA (age, 73±9 years;42.2% female) were enrolled in this study. 30 healthy participants from preliminary studies with unremarkable cardiac findings on CMR were included as control group (age, 53±12 years; 46.7% female).

The GCA patients underwent baseline CMR after a median of 22 days. 24/45 patients (53%) were diagnosed with large-vessel involvement; 21/45 patients (46.7%) had only cranial vessel involvement. Laboratory results showed elevated inflammatory parameters at baseline: C-reactive protein levels (CRP) 23.4±28.7 mg/L (normal <5 mg/L), erythrocyte sedimentation rate 52.1±26.4 mm/h (normal <20 mm/h), and leukocyte count 9.6±3.2 ×10⁹/L (normal 4.0–10.0 ×10⁹/L). The median number of cardiovascular risk factors per patient was 1(IQR, 0–2). The most frequent cardiovascular risk factors were hypertension (21/45; 46.7%), smoking (11/45; 24.4%), and obesity (11/45; 24.4%). Diabetes was observed in 10/45 patients (22.2%) and atherosclerosis in 11/45 (24.4%). In addition, 2/45 patients (4.4%) had previously known peripheral artery disease. Previously known CAD was documented in 7 of 45 patients (15.6%). Among these, five patients had undergone prior therapeutic procedures: four had prior PCI/coronary stenting, and one had prior CABG. None of the patients had previously known inflammatory myocardial disease. One of the patients reported acute symptoms of cardiovascular disease before baseline CMR (2.2%). During follow-up of a median of 22.5 months (IQR, 19.1–30.5), cardiovascular events were recorded in 6/45 patients (13.3%). These included stroke (1/6 patients), non-ST-elevation myocardial infarction requiring coronary artery bypass grafting (1/6 patients), new episodes of atrial fibrillation (2/6 patients), hospitalization for cardiac decompensation in valvular heart disease and significant coronary artery disease requiring complex PCI (1/6 patients), and newly detected subsegmental small myocardial infarction with a predominantly embolic pattern in a clinically asymptomatic patient (1/6; see also Fig. 3).

Relevant baseline demographic and clinical parameters are given in Table 1. Patients with GCA were significantly older than healthy controls (73±9 vs. 53±12 years; p<0.001). No significant differences were observed in body mass index (26.1±5.0 vs. 25.6±5.4 g/m²; p=0.683) or body surface area (1.9±0.2 vs. 2.0±0.2 m²; p=0.250) between both groups; see Table 2**.**

**Table 2:** Clinical and baseline cardiovascular magnetic resonance imaging (CMR) characteristics in patients with giant cell arteritis (GCA) compared to healthy controls

	GCA (n=45)	Controls (n=30)	p-value
*Clinical parameters*			
Age (y)	73±9	53±12	**<0.001**
Sex (female)	19/45 (42.0%)	14/30 (46.7%)	0.709
Body mass index (kg/ m²)	26.1±5.0	25.6±5.4	0.683
Body surface area (m²)	1.89±0.24	1.96±0.22	0.250
Heart rate (bpm)	68±13	65±11	0.335
*Cardiovascular magnetic resonance*			
LV ejection fraction (%)	63±9	61±6	0.380
LV EDV/BSA (mL/m²)	74.2±21.3	80.3±15.4	0.188
LV SV/BSA (mL/m²)	45±13	48±10	0.221
LV mass/BSA (g/m²)	55±17	45±8	**0.031**
RV EF (%)	55±7	52±7	0.086
RV EDV/BSA (mL/m²)	74±27	81±16	0.262
Global circumferential strain	-24.3±5.0	-24.1±3.2	0.861
Global longitudinal strain	-20.7±4.7	-21.8±3.2	0.247
Wall motion abnormality (Hypo-/akinesia)	6/45 (13.3%)	0/30 (0.0%)	0.074
Pericardial effusion (> 9 mm), n (%)	1/45 (2.2%)	0/30 (0.0%)	1.000
Visual edema on T2 STIR (n, %)	3/45 (6.7%)	0/30 (0.0%)	0.239
− Myocardial	1/3 (33.3%)	-	-
− Pericardial	2/3 (66.7%)	-	-
T2 relaxation times (ms)	54±3	54±2	0.235
Elevated T2 relaxation times	8/45 (17.8%)	0/30 (0.0%)	**0.006**
Late gadolinium enhancement (LGE)	12/45 (26.7%)	0/30 (0.0%)	**<0.001**
− Ischemic	5/12 (41.7%)	-	-
− Non-ischemic	5/12 (41.7%)	-	-
− Pericardial	2/12 (16.7%)	-	-
T1 relaxation times (ms)	987±29	968±22	**0.003**
Elevated T1 relaxation times	10/45 (22.2%)	0/30 (0.0%)	**0.001**
Extracellular volume fraction (%)	27.3±3.8	25.2±2.1	**0.003**
Elevated extracellular volume fraction	8/45 (17.8%)	0/30 (0.0%)	**0.006**

Data are numbers (%) of cases or means ± standard deviation. *LV* left ventricular, *EDV* end-diastolic volume, *SV* stroke volume, *ED* mass, end-diastolic mass, *CO* cardiac output, *RV* right ventricular, *BSA* body surface area, *LGE* late gadolinium enhancement, *STIR* short tau inversion recovery

### 3.2 Myocardial function and volumes

CMR revealed no significant difference in left ventricular ejection fraction (LVEF) between GCA patients and healthy controls (63±9 vs. 61±6%; p=0.380); 3/45 patients (6.7%)had an LVEF <50%. Regional wall motion abnormalities (focal hypokinesia) were seen in 6/45 patients (13.3%). Indexed left ventricular volumes did not differ from healthy controls (e.g., LV EDV/BSA 74.2±21.3 vs. 80.3±15.4 ml/m², p=0.188). Indexed left ventricular mass was significantly higher in patients compared to healthy controls (55±17 vs. 45±8 g/m²; p=0.031).

CMR revealed no significant differences in myocardial global circumferential strain (−24.3±5.0 vs. −24.1±3.3%; p=0.861) and myocardial global longitudinal strain (−20.7±4.7 vs. −21.8±3.3%; p=0.247) between GCA patients and healthy controls. See Table 2 for detailed parameters.

### 3.3 Cardiac edema/inflammation and pericardial effusion

**Fig. 1:**
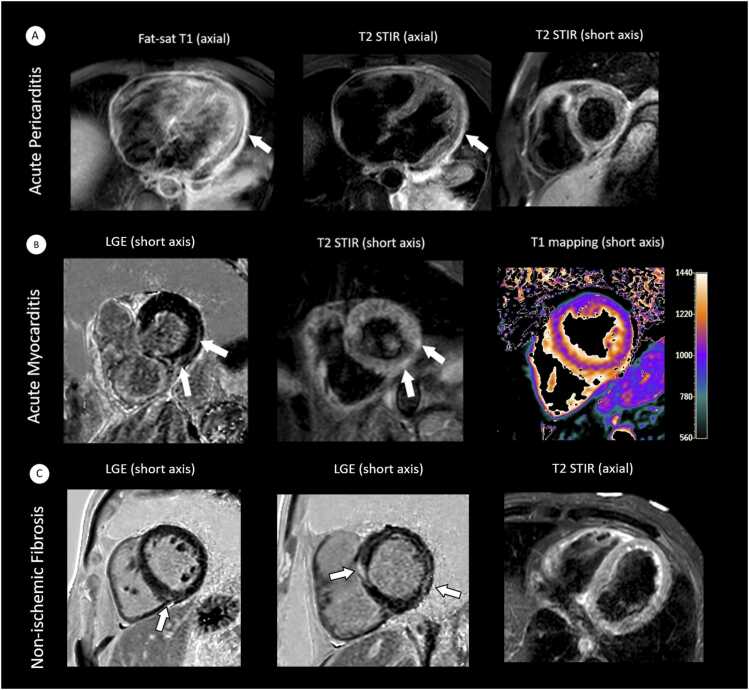
Exemplary cardiovascular magnetic resonance findings of different patients with first diagnosis of giant cell arteritis and active or post-inflammatory cardiac disease. A, 63-year-old male patient with active pericarditis (arrows show circular pericardial thickening and hyperenhancement on contrast-enhanced fat-sat T1 images with corresponding pericardial edema on T2 STIR). B, 79-year-old female patient with active myocarditis (arrows show subepicardial hyperenhancement on LGE with corresponding focal edema on T2 STIR [Short-Tau-Inversion-Recovery] and elevated myocardial native T1 relaxation times). C, 60-year-old male patient with subepicardial and midmyocardial LGE of the lateral and anterior wall without associated edema; this non-ischemic pattern could be compatible with post-inflammatory fibrosis. *LGE* late gadolinium enhancement

**Fig. 2:**
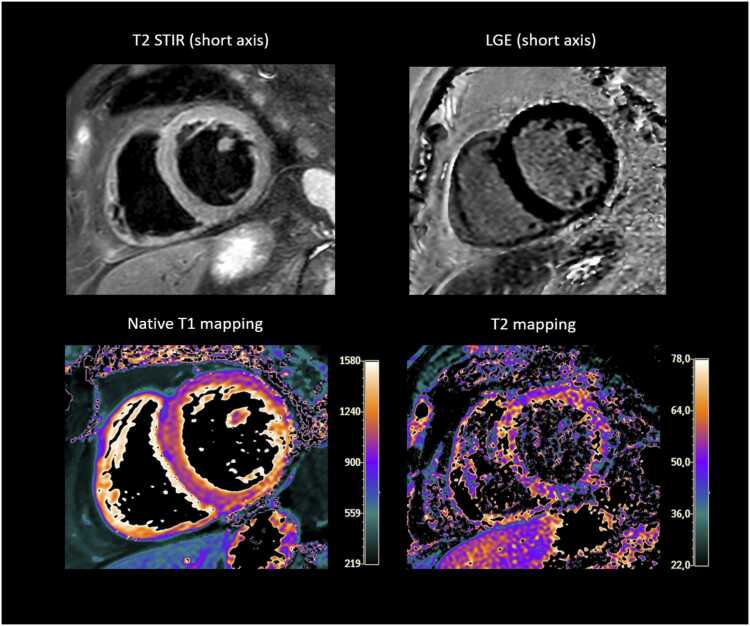
Eighty-one-year-old male patient with combined elevation of myocardial mapping parameters (mean T1 relaxation time: 1055 ms, reference cut off ≥1000 ms; mean T2 relaxation time: 60 ms, reference cut off ≥55.9 ms) indicating diffuse myocardial edema. No focal abnormalities were observed on T2 STIR or LGE. *LGE* late gadolinium enhancement, *STIR* short-tau-inversion-recovery

Visual edema on T2 STIR was seen in 3/45 patients (6.7%), compatible with acute myocarditis and active pericarditis (see Fig. 1). About 8/45 patients (17.8%) had elevated myocardial T2 relaxation times compared to the center-specific cut-off (see Fig. 2). Compared to healthy controls no significant differences were found in global T2 relaxation times (54±3 vs. 54±2 ms; p=0.235); see Table 2. 4/45 patients (8.9%) had combined elevation of T1 and T2 relaxation times indicating diffuse edema. Pericardial effusion was present in 5/45 cases (11.1%; <9 mm in 4/45 [8.9%], > 9 mm in 1/45 [2.2%] cases). No significant associations were found between myocardial tissue parameters and laboratory inflammation markers (e.g., T1 vs. CRP: r = –0.18, p=0.229; see Table S2).

### 3.4 Myocardial fibrosis

Myocardial T1 mapping showed elevated native T1 relaxation times in 10/45 patients (22.2%) and elevated ECV in 8/45 cases (17.8%) according to center-specific reference values. LGE was present in 12/45 patients (26.7%) with ischemic distribution in 5/12 (41.7%), non-ischemic possible inflammatory distribution in 5/12 (41.7%), and pericardial distribution in 2/12 (16.7%). About 4/45 patients (8.9%) had an unspecific subtle enhancement at the RV insertion. Myocardial tissue characterization revealed significantly elevated native T1 values (987±29 vs. 968±22 ms; p=0.003) and ECV (27.3±3.8 vs. 25.2±2.1%; p=0.003) in patients compared to healthy controls (see Fig. 4). Pearson correlation analysis did not reveal any significant associations between the number of cardiovascular risk factors per patient and any of the tissue parameters (T1 mapping: r=–0.071, p=0.643; T2 mapping: r=(-0.131), p=0.392; ECV: r=(-0.019), p=0.899); see Table S2.

**Fig. 3:**
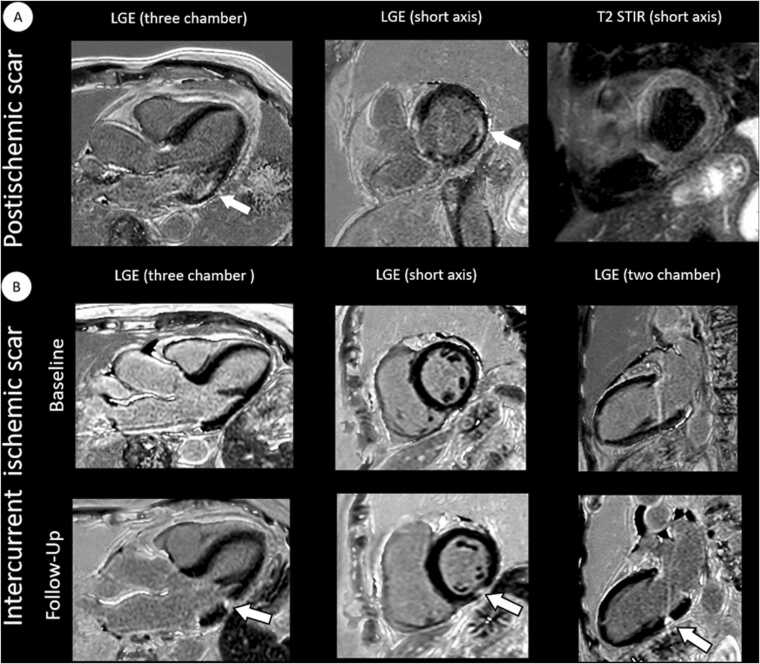
Exemplary cardiovascular magnetic resonance (findings of different patients with first diagnosis of giant cell arteritis and post-ischemic myocardial scarring). A, 76-year-old male patient with post-ischemic scar of the basal lateral wall (arrows show subendocardial hyperenhancement on LGE). B, 79-year-old female patient with giant cell arteritis and no LGE lesions at baseline. Follow-up imaging revealed a newly developed, subsegmental, transmural LGE in the midventricular inferior segment, consistent with a small myocardial infarction with a primarily embolic pattern. Both patients were clinically asymptomatic and had no history of cardiac events. *LGE* late gadolinium enhancement

**Fig. 4:**
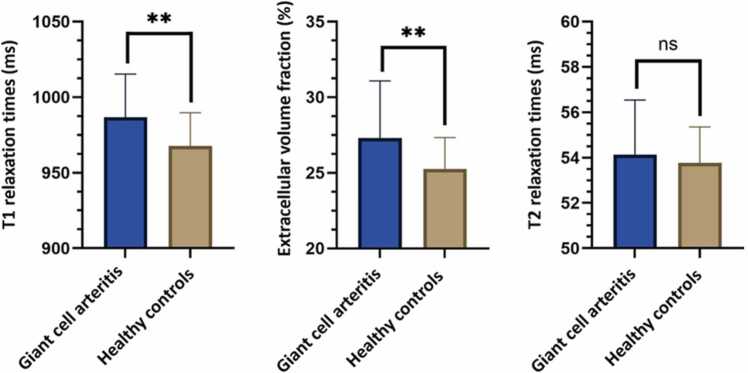
Bar graphs showing T1 mapping, extracellular volume fraction values and T2 mapping in patients with giant cell arteritis compared to healthy controls. Data are presented as mean ± standard deviation. *** = p<0.001, ** = p< 0.05, ns = non significant

### 3.5 Cardiovascular magnetic resonance diagnosis

On baseline CMR, 29/45 (64.4%) of GCA patients demonstrated unremarkable (15/29, 51.7%) or nonspecific findings (14/29, 48.3%). Of patients with nonspecific findings, myocardial alterations were as follows: unspecific LGE at RV insertion (4/14, 28.6%), isolated elevation of T1 relaxation times (6/14, 42.9%), isolated elevation in T2 relaxation times (4/14, 28.6%). About 3/45 (6.7%) had findings consistent with active inflammatory cardiac disease: one patient had acute myocarditis, and two patients were diagnosed with active pericarditis; see Fig. 1. 4/45 (8.9%) patients had combined elevation of T1 and T2 relaxation times (patients without known coronary artery disease and without LGE lesions), principally compatible with diffuse myocardial inflammation. Exactly 5/12 (41.7%) patients displayed LGE with non-ischemic pattern; except for the patient with active myocarditis described above, the findings were compatible with post-inflammatory fibrosis. 5/12 (41.7%) patients showed LGE with ischemic distribution indicating post-ischemic scars (see Fig. 3). Of these, 3/5 (60%) had no previously known CAD.

### 3.6 Cardiovascular magnetic resonance follow-up

**Fig. 5:**
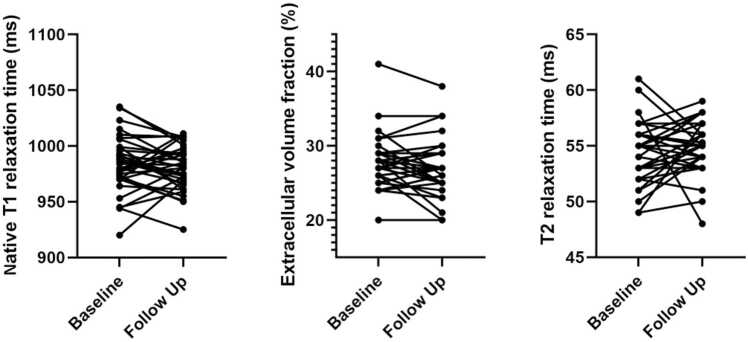
Paired dot plots of myocardial tissue parameters in patients with giant cell arteritis at baseline and follow-up

Follow-up CMR was prospectively planned per study protocol for all enrolled patients at 6 months after baseline. Overall, 35/45 patients (77.8%) underwent follow-up CMR after a median of 168 days (IQR, 154–190). Non-participation was due to logistical reasons, patient preference, or newly occurring MRI contraindications. No significant changes were observed in cardiac function, strain, or tissue characteristics on follow-up (e.g., LVEF: 63±9% vs. 62±8%, p=0.172; myocardial global longitudinal strain: −21.1±4.7 vs. −19.8±4.2%, p=0.084; see Table 3. Myocardial tissue markers, including native T1 (984±25 vs. 980±20 ms, p=0.397), T2 (54±3 vs. 55±2 ms, p=0.140), and ECV values (27.4±3.6% vs. 26.8±3.8%, p=0.245), did not significantly change on follow-up; see Fig. 5. One asymptomatic GCA patient had a new focal post-ischemic LGE in the follow-up period compatible with a silent subsegmental myocardial infarction (embolic pattern); see Fig. 3.

**Table 3:** Comparison of cardiovascular magnetic resonance imaging (CMR) parameters in patients with GCA at baseline and at follow-up CMR

	Baseline (n=35)	Follow-up (n=35)	p-value
Heart rate (bpm)	69±14	68±11	0.536
LV ejection fraction (%)	63±9	62±8	0.172
LV EDV/BSA (mL/m²)	74±16	71±12	0.144
LV SV/BSA (mL/m²)	45±14	46±13	0.417
LV mass/BSA (g/m²)	54±18	55±12	0.621
Intraventricular septal thickness, segment 3 (mm)	10.6±3.4	10.0±2.7	0.144
Right ventricular ejection fraction (%)	55±7	55±7	0.880
RV EDV/BSA (ml/m²)	76±30	73±20	0.503
Global circumferential strain	-24.58±5.23	-23.62±4.56	0.114
Global longitudinal strain	-21.06±4.68	-19.78±4.16	0.084
Wall motion abnormality (Hypo-/akinesia)	4/35 (11.4%)	4/35 (11.4%)	1.000
Pericardial effusion (> 9 mm), n (%)	1/35 (2.9%)	0/35 (0.0%)	1.000
Visual edema on T2 STIR	2/35 (5.7%)	1/35 (2.9%)	0.500
− Myocardial	0/2 (0.0%)	0/1 (0.0%)	1.000
− Pericardial	2/2 (100.0%)	1/1 (100.0%)	1.000
T2 relaxation times (ms)	54±3	55±2	0.140
Elevated T2 relaxation times	6/35 (17.1%)	9/35 (25.7%)	0.453
Late gadolinium enhancement, presence	10/35 (28.6%)	11/35 (31.4%)	0.688
− Ischemic	4/10 (40.0%)	5/11 (45.5%)	1.000
− Non-ischemic	4/10 (40.0%)	4/11 (36.4%)	1.000
− Pericardial	2/10 (20.0%)	2/11 (18.2%)	1.000
T1 relaxation times (ms)	984±25	980±20	0.397
Elevated T1 relaxation times	6/35 (17.1%)	7/35 (20.0%)	1.000
Extracellular volume fraction (%)	27.4±3.6	26.8±3.8	0.245
Elevated extracellular volume fraction	6/35 (17.1%)	5/35 (14.3%)	1.000

Data are numbers (%) of cases or means ± standard deviation. *LV* left ventricular, *EDV* end-diastolic volume, *SV* stroke volume, *CO* cardiac output, *RV* right ventricular, *BSA* body surface area, *LGE* late gadolinium enhancement, *STIR* short tau inversion recovery

### 3.7 Disease phenotype of giant cell arteritis

Patients with large-vessel involvement did not differ in age (74±9 vs. 73±10 years, p=0.772) compared to those without. The proportion of male patients was higher in the large-vessel group (75% vs. 38%, p=0.017). Patients with large-vessel involvement had significantly higher myocardial ECV values (28.7±4.2% vs. 25.8±2.5%, p=0.008), but no significant difference in native T1 (987± 32 vs. 987±26 ms, p=0.959) and T2 values (54±3 vs. 55±2 ms, p=0.697). Other CMR parameters (e.g., ejection fraction, volumes) did not differ significantly (Table S1).

## 4 Discussion

In this prospective study, CMR revealed signs of cardiac involvement in patients with newly diagnosed GCA, including diffuse myocardial abnormalities suggestive of chronic or post-inflammatory disease. These findings may reflect a less recognized form of subclinical inflammatory myocardial involvement and appear consistent with post-inflammatory fibrotic changes. Notably, patients with large-vessel involvement showed higher ECV while native T1 and T2 did not differ, indicating a more subtle difference limited to extracellular volume expansion, supporting the hypothesis of immunophenotypic divergence within the disease spectrum. Moreover, myocarditis and pericarditis were diagnosed in individual cases at the time of GCA diagnosis, indicating possible overlap with inflammatory cardiac conditions. Additionally, post-ischemic scars were observed, supporting the reported increased risk of coronary artery disease in GCA. The patient with intercurrent ischemic scar had no known prior cardiac disease or history of arrhythmia (including atrial fibrillation) and was not on anticoagulation at the time of CMR or during the available follow-up. In our cohort, approximately 27% of patients exhibited LGE, an unexpectedly high prevalence comparable to that reported in the other large vessel vasculitis known to affect the heart, such as Takayasu arteritis [16]. LGE was generally not accompanied by elevated T1 or T2, suggesting a more chronic or post-inflammatory fibrotic remodeling process rather than an acute inflammation [17]. In a subset of the cohort, the observed subepicardial LGE pattern, localized to the basal to mid-inferolateral segments, resembled that seen in immune-mediated myocarditis and has been histologically associated with lymphocytic or macrophage-rich infiltrates [18]. Although acute myocarditis is rarely reported in GCA, subclinical myocardial involvement may be more common [2]. However, while LGE is highly specific for focal fibrosis (or necrosis in acute settings), it is limited in detecting diffuse or early-stage myocardial changes [19].

Interestingly, native myocardial T1 and ECV values were significantly higher in GCA patients compared to controls, while T2 mapping showed no significant differences, indicating the presence of diffuse myocardial fibrosis. Although the underlying immunopathogenesis differs substantially, our findings in GCA mirror those from a previous study on ANCA-associated vasculitis, where also elevated T1 and ECV but not T2 values, were reported [20]. In contrast, a study on rheumatoid arthritis showed a significant increase in T2 relaxation times, along with elevated native T1 and ECV values suggesting that disease-specific inflammatory patterns may lead to distinct myocardial tissue alterations detectable by CMR [21]. In our study, four patients had concurrent elevation of T1 and T2 values, consistent with diffuse myocardial edema and potentially reflecting subclinical diffuse myocarditis. Notably, these individuals lacked overt cardiac symptoms.

The detection of active myocarditis and pericarditis in individual GCA patients (7% of our cohort) may justify closer cardiovascular surveillance. In line with prior reports describing myocarditis in GCA as an uncommon but serious presentation, our cohort included one patient with myocarditis [3]. This underscores that although myocarditis is rare, it should be considered in symptomatic patients and can be assessed non-invasively by CMR. The patient became symptomatic due to headache and jaw pain, which was primarily distributed to GCA, but may also include atypical presentation of myocarditis. One of the cases with pericarditis presented with chest pain and shoulder pain, while the other case presented only with ophthalmological GCA-related symptoms. In our clinical practice, these findings have strengthened interdisciplinary collaboration and led us to consider a combined vascular MRA–CMR protocol in selected newly diagnosed GCA patients (e.g., with large-vessel involvement, elevated cardiac biomarkers, or symptoms suggestive of myocardial involvement). Patients with large-vessel involvement demonstrated significantly higher myocardial ECV. In the absence of T2 elevation, increased ECV likely reflects chronic interstitial expansion due to diffuse fibrosis. Unlike ECV, native T1 captures both intra- and extracellular compartments and is more susceptible to physiological confounders such as heart rate [4]. These findings align with emerging evidence suggesting that the pattern and extent of vascular involvement in GCA may reflect distinct immunophenotypic disease subsets. GCA of the cranial vessels is associated with ischemic ophthalmologic complications 11, 22, 23. In contrast, large-vessel involvement appears to confer a lower risk for ischemic ocular events [24]. Although the mechanisms underlying this divergence remain unclear, proposed explanations include a higher systemic inflammatory burden in large-vessel GCA, IL-6–mediated neoangiogenesis contributing to tissue protection, and differential cell and cytokine-driven immunopathology [25]. These observations raise important questions about the pathophysiological link between large-vessel involvement and myocardial changes. One possibility is that large-vessel involvement associated pronounced systemic inflammatory response might predispose to parallel myocardial involvement and indicate a shared immunophenotype. Alternatively, local inflammatory processes in the aorta or its major branches may exert indirect effects on the myocardium.

The extent to which glucocorticoid therapy modulates systemic and cardiac involvement in GCA remains unclear. Multimodal vascular imaging studies have demonstrated that anti-inflammatory treatment, particularly glucocorticoids, rapidly reduces the sensitivity of imaging modalities to detect vascular inflammation 26, 27, 28. However, active vascular inflammation may persist even in patients considered to be in clinical remission 29, 30. This discrepancy is likely attributable to complex molecular mechanisms, including dysregulation of fibroblasts and stromal cells, which contribute to chronic inflammation and vascular remodeling [31].

The absence of dynamic changes on follow-up CMR further supports the interpretation of a chronic, stable myocardial phenotype. We observed no correlation between myocardial T1, T2, or ECV values and systemic inflammatory markers such as CRP, suggesting that the diffuse myocardial tissue alterations reflect residual post-inflammatory remodeling rather than active disease. Even without symptoms, myocardial tissue changes in our study raise questions about their role as early indicators of increased cardiovascular risk. In other inflammatory conditions, similar findings, such as non-ischemic LGE and elevated T1 values were linked to impaired heart function, arrhythmias, and poor long-term outcomes [32]. Further investigations should integrate clinical phenotyping and cytokine profiling to clarify the mechanisms of cardiac involvement in GCA and determine if imaging biomarkers predict long-term cardiovascular outcomes. Future studies should assess longer-term clinical outcomes to determine whether CMR parameters provide independent prognostic value in GCA.

## 5 Limitations

Several limitations have to be discussed. First, patients underwent baseline CMR shortly after their initial diagnosis of GCA but after the immediate initiation of glucocorticoid therapy; rapidly regressing inflammatory changes may have been underestimated. The sample size was limited, particularly for the GCA subgroups with cranial-only disease and those with large-vessel involvement. Accordingly, the largely non-significant subgroup differences should be interpreted cautiously and do not exclude phenotype-specific myocardial involvement. Larger, phenotype-stratified multicenter cohorts are needed to confirm these findings and clarify potential links between vascular and myocardial inflammation. Second, no histologic or molecular validation of myocardial changes was performed; therefore, the exact causality or pathogenesis underlying the described cardiac findings cannot be determined. There is an age disparity between patients with GCA and the healthy control group because CMR scans of entirely healthy patients in this age group are uncommon. Incomplete follow-up (35/45 patients) may introduce bias. No pre-diagnostic CMR was available, so it remains unclear whether LGE reflects pre-existing scars or GCA-related change; longitudinal CMR studies are needed. Since some GCA patients present with cardiovascular risk factors or pre-existing coronary artery disease, it is challenging to fully distinguish myocardial changes attributable to GCA from those resulting from other cardiovascular conditions. Furthermore, no pre-diagnostic or historical CMR data were available for comparison. Consequently, we cannot determine whether LGE findings represent pre-existing myocardial scars or GCA-related alterations. Future longitudinal studies including screening for pre-treatment imaging or serial CMR follow-up are warranted to clarify the temporal relationship between GCA onset and myocardial fibrosis. We used a retrospective historical control group, which may introduce selection bias and residual confounding; results should be interpreted cautiously and confirmed in prospectively matched cohorts.

## 6 Conclusion

Patients with newly diagnosed GCA exhibit signs of cardiac involvement—such as myocarditis, pericarditis and diffuse (post) inflammatory myocardial alterations, as well as post-ischemic fibrosis—suggesting that cardiac involvement may be more common in GCA than previously appreciated.

## Author contributions

**Annemarie Proff:** Writing – review & editing, Writing – original draft, Visualization, Project administration, Methodology, Investigation, Formal analysis, Data curation, Conceptualization. **Simon M. Petzinna:** Writing – review & editing, Validation, Supervision, Project administration, Methodology, Investigation, Data curation, Conceptualization, Writing – original draft. **Lena Kreis:** Writing – review & editing, Data curation. **Sophie-Marie Kirch:** Writing – review & editing, Investigation, Data curation. **Taraneh Aziz-Safaie:** Writing – review & editing, Visualization. **Narine Mesropyan:** Writing – review & editing. **Dmitrij Kravchenko:** Writing – review & editing. **Anja Winklbauer:** Writing – review & editing, Data curation. **Tatjana Dell:** Writing – review & editing. **Claus C. Pieper:** Writing – review & editing. **Daniel Kuetting:** Writing – review & editing. **Julian A. Luetkens:** Writing – review & editing, Validation, Supervision, Project administration, Methodology, Investigation, Funding acquisition, Conceptualization. **Schafer Valentin S.Schäfer:** Writing – review & editing, Validation, Supervision, Project administration, Methodology, Funding acquisition, Conceptualization. **Alexander Isaak:** Writing – review & editing, Writing – original draft, Visualization, Validation, Supervision, Software, Resources, Project administration, Methodology, Investigation, Funding acquisition, Formal analysis, Data curation, Conceptualization.

## Declaration of competing interests

The authors declare that they have no known competing financial interests or personal relationships that could have appeared to influence the work reported in this paper.

## Data Availability

The datasets used and/or analyzed during the current study is not publicly available, but is available from the corresponding author upon reasonable request.
