# Supplementary material for: Cardiovascular magnetic resonance detects subclinical cardiac involvement in giant cell arteritis
Source: J Cardiovasc Magn Reson. 2026 Apr 27;28(1):102740. doi: 10.1016/j.jocmr.2026.102740 (PMC13241631; doi:10.1016/j.jocmr.2026.102740)

# SUPPLEMENTARY DATA

**Table S1:** Subgroup analysis comparing patients with and without large-vessel involvement of giant cell arteritis.

| Parameter | Large Vessel GCA (n=24) | Non-Large Vessel GCA (n=21) | P-Value |
| --- | --- | --- | --- |
| *Clinical parameter* |  |  |  |
| Age (years) | 74±9 | 73±9 | 0.772 |
| Sex (female) | 6/24(25.0%) | 13/21(61.9%) | **0.017** |
| Body mass index (kg/m²) | 25.8±3.8 | 26.5±6.2 | 0.675 |
| Body surface area (m²) | 1.93±0.21 | 1.85±0.26 | 0.268 |
| OMERACT GCA ultrasonography score | 1.2±0.3 | 1.2±0.4 | 0.575 |
| *Cardiovascular MRI parameters* |  |  |  |
| LV ejection fraction (%) | 61±9 | 64±9 | 0.220 |
| LV EDV/BSA (ml/m²) | 75.8±25.9 | 72.4±14.9 | 0.589 |
| LV SV/BSA (ml/m²) | 44±15 | 46±9 | 0.702 |
| LV mass/BSA (g/m²) | 56.2±16.4 | 52.5±17.1 | 0.457 |
| Intraventricular septal thickness, segment 3 (mm) | 12.9±3.4 | 11.6±2.9 | 0.162 |
| Right ventricular ejection fraction (%) | 53±7 | 57±7 | 0.117 |
| RV EDV/BSA (ml/m²) | 76±29 | 72±24 | 0.659 |
| Late gadolinium enhancement presence | 7/24(29.2%) | 5/21(23.8%) | 0.730 |
| - Ischemic | 3/7(42.9%) | 2/5(40.0%) | 1.000 |
| - Non-ischemic | 3/7(42.9%) | 2/5(40.0%) | 1.000 |
| - Pericardial | 1/7(14.3%) | 1/5(20.0%) | 1.000 |
| Visual edema on T2 STIR | 2/24(8.3%) | 1/21(4.8%) | 1.000 |
| - Myocardial | 1/2(50.0%) | 0/1(0.0%) | 1.000 |
| - Pericardial | 1/2(50.0%) | 1/1(100.0%) | 1.000 |
| T1 Mapping (ms) | 986±32 | 987±26 | 0.959 |
| T2 Mapping (ms) | 54±3 | 53±2 | 0.210 |
| Extracellular Volume fraction (%) | 28.7±4.2 | 25.8±2.5 | **0.008** |

LV, left ventricular; EDV, end-diastolic volume; SV, stroke volume; ED Mass, end-diastolic mass; CO Index, cardiac output indexed to body surface area; RV, Right ventricular; BSA, body surface area; LGE, Late Gadolinium Enhancement; STIR, short tau inversion recovery.

**Table S2:** Pearson correlation between cardiac tissue parameters, laboratory inflammation markers and amount of cardiovascular risk factors.

|  | T1 mapping | T2 mapping | Extracellular volume fraction (%) | CRP (mg/L) | Leukocytes (10⁹/L) | Number of cardiovascular risk factors per person |
| --- | --- | --- | --- | --- | --- | --- |
| T1 Mapping | – | 0.545 (p<0.001) | 0.361  (p=0.015) | –0.183 (p=0.229) | –0.093  (p=0.545) | –0.071  (p=0.643) |
| T2 Mapping | 0.545 (p<0.001) | – | 0.326  (p=0.029) | 0.088 (p=0.566) | –0.038  (p=0.805) | -0.131  (p=0.392) |
| Extracellular volume fraction (%) | 0.361 (p=0.015) | 0.326  (p=0.029) | – | –0.003 (p=0.982) | 0.028  (p=0.854) | –0.019  (p=0.899) |
| CRP (mg/L) | –0.183 (p=0.229) | 0.088 (p=0.566) | –0.003  (p=0.982) | – | 0.104  (p=0.495) | –0.090  (p=0.556) |
| Leukocytes (10⁹/L) | –0.093 (p=0.545) | –0.038 (p=0.805) | 0.028  (p=0.854) | 0.104 (p=0.495) | – | –0.186  (p=0.221) |
| Number of cardiovascular risk factors per person | –0.071  (p=0.643) | –0.131  (p=0.392) | –0.019  (p=0.899) | –0.090  (p=0.556) | –0.186  (p=0.221) | – |

**Table S3**: Clinical and baseline cardiovascular magnetic resonance imaging (CMR) characteristics in patients with large vessel giant cell arteritis (GCA) compared to healthy controls.

|  | Large-vessel GCA (n= 24) | Controls (n = 30) | P-value |
| --- | --- | --- | --- |
| *Clinical parameters* |  |  |  |
| Age (years) | 74±9 | 53±12 | **<0.001** |
| Sex (female) | 6/24(25.0%) | 14/30(47.0%) | 0.087 |
| Body mass index (kg/ m²) | 25.8±3.8 | 25.6±5.4 | 0.877 |
| Body surface area (m²) | 1.93±0.21 | 1.96±0.22 | 0.662 |
| *Cardiovascular magnetic resonance* |  |  |  |
| LV ejection fraction (%) | 61±9 | 61±6 | 0.972 |
| LV EDV/BSA (ml/m²) | 75.8±25.9 | 80.3±15.4 | 0.464 |
| LV SV/BSA (ml/m²) | 44±15 | 48±10 | 0.271 |
| LV mass/BSA (g/m²) | 56±16 | 45±8 | **0.009** |
| RV EF (%) | 53±7 | 52±7 | 0.483 |
| RV EDV/BSA (ml/m²) | 75±29 | 81±16 | 0.419 |
| Myocardial Global Circumferential Strain | -24.33±5.50 | -24.10±3.17 | 0.851 |
| Myocardial Global Longitudinal Strain | -20.15±4.50 | -21.81±3.15 | 0.120 |
| Endocardial Global Circumferential Strain | -36.90±9.24 | -35.44±5.29 | 0.503 |
| Endocardial Global Longitudinal Strain | -22.76±5.13 | -24.61±4.08 | 0.148 |
| Wall motion abnormality (Hypo-/akinesia) | 4/24(16.7%) | 0/30(0.0%) | **0.034** |
| Pericardial effusion (> 9mm), (n, %) | 0/24 (0.0%) | 0/30(0.0%) | 1.000 |
| Visual edema on T2 STIR (n, %) | 2/24(8.3%) | 0/30(0.0%) | 0.193 |
| - Myocardial | 1/2 (50.0%) | 0/0(0.0%) | 1.000 |
| - Pericardial | 1/2(50.0%) | 0/0(0.0%) | 1.000 |
| T2 relaxation times (ms) | 54±3 | 54±2 | 0.766 |
| Elevated T2 relaxation times | 7/24(29.2%) | 0/30(0.0%) | **0.002** |
| Late gadolinium enhancement (LGE) | 7/24(29.2%) | 0/30(0.0%) | **0.002** |
| Ischemic | 3/7(42.9%) | 0/0(0.0%) | 0.082 |
| - Non-ischemic | 3/7(42.9%) | 0/0(0.0%) | 0.082 |
| - Pericardial | 1/7(14.3%) | 0/0(0.0%) | 0.444 |
| T1 relaxation times (ms) | 986±32 | 968±22 | **0.003** |
| Elevated T1 relaxation times | 6/24(25.0%) | 0/30(0.0%) | **0.013** |
| Extracellular volume fraction (%) | 28.7±4.2 | 25.2±2.1 | **<0.001** |
| Elevated extracellular volume fraction | 7/24(29.2%) | 0/30(0%) | **0.002** |

LV, left ventricular; EDV, end-diastolic volume; SV, stroke volume; ED mass, end-diastolic mass; CO, cardiac output; RV, right ventricular; BSA, body surface area; LGE, late gadolinium enhancement; STIR, short tau inversion recovery.

**Table S4:** Comparison of cardiovascular magnetic resonance imaging (CMR) parameters in patients with large-vessel GCA at baseline and at follow-up CMR.

|  | Baseline (n = 20) | Follow-up (n = 20) | P-value |
| --- | --- | --- | --- |
| Heart rate (bpm) | 69±15 | 68±10 | 0.779 |
| LV ejection fraction (%) | 63±9 | 62±7 | 0.657 |
| LV EDV/BSA (ml/m²) | 74±26 | 71±16 | 0.351 |
| LV SV/BSA (ml/m²) | 43±16 | 46±12 | 0.321 |
| LV mass/BSA (g/m²) | 56±17 | 57±13 | 0.554 |
| Intraventricular septal thickness, segment 3 (mm) | 10.9±3.3 | 10.6±3.1 | 0.501 |
| Right ventricular ejection fraction (%) | 52±7 | 56±7 | **0.006** |
| RV EDV/BSA (ml/m²) | 75±30 | 73±18 | 0.917 |
| Myocardial Global Circumferential Strain | -24.5±5.6 | -23.5±4.3 | 0.319 |
| Myocardial Global Longitudinal Strain | -20.6±4.5 | -20.0±3.6 | 0.583 |
| Endocardial Global Circumferential Strain | -36.8±9.1 | -35.6±6.2 | 0.492 |
| Endocardial Global Longitudinal Strain | -23.1±5.2 | -22.5±4.0 | 0.645 |
| Wall motion abnormality (Hypo-/akinesia) | 4/20(20.0%) | 2/20(10.0%) | 0.500 |
| Pericardial effusion (> 9mm), n (%) | 0/20(0%) | 0/20(0%) | 1.000 |
| Visual edema on T2 STIR | 1/20 (5%) | 1/20(5.0%) | 1.000 |
| - Myocardial | 0/1(0%) | 0/1(0%) | 1.000 |
| - Pericardial | 1/1(100.0%) | 1/1(100.0%) | 1.000 |
| T2 relaxation times (ms) | 54±3 | 55±3 | 0.174 |
| Elevated T2 relaxation times | 5/20(25.0%) | 4/20(20.0%) | 1.000 |
| Late gadolinium enhancement, presence | 5/20(25.0%) | 6/20(30.0%) | 1.000 |
| - Ischemic | 2/5(40.0%) | 3/6(50.0%) | 1.000 |
| - Non-ischemic | 2/5(40.0%) | 2/6(33.3%) | 1.000 |
| - Pericardial | 1/5(20.0%) | 1/6(16.7%) | 1.000 |
| T1 relaxation times (ms) | 983±28 | 982±21 | 0.731 |
| Elevated T1 relaxation times | 4/20(20.0%) | 4/20(20.0%) | 1.000 |
| Extracellular volume fraction (%) | 28.3±4.0 | 27.2±4.1 | 0.130 |
| Elevated extracellular volume fraction | 4/20(20.0%) | 5/20(25.0%) | 1.000 |

LV, left ventricular; EDV, end-diastolic volume; SV, stroke volume; CO, cardiac output; RV, right ventricular; BSA, body surface area; LGE, late gadolinium enhancement; STIR, short tau inversion recovery.

**Figure S1.** Exemplary cardiovascular magnetic resonance findings in patients with newly diagnosed giant cell arteritis (GCA) and subclinical pericardial involvement with follow-up imaging. A, 63-year-old male patient with subtle pericardial edema and non-ischemic pericardial hyperenhancement. B, 75-year-old female patient with similarly discrete pericardial abnormalities, showing mild pericardial edema and non-ischemic pericardial hyperenhancement at baseline. Both patients were clinically asymptomatic with respect to pericarditis at the time of CMR. In follow-up CMR of the two pericarditis patients demonstrating improvement of pericardial findings with reduced edema and decreased extent/intensity of pericardial enhancement.


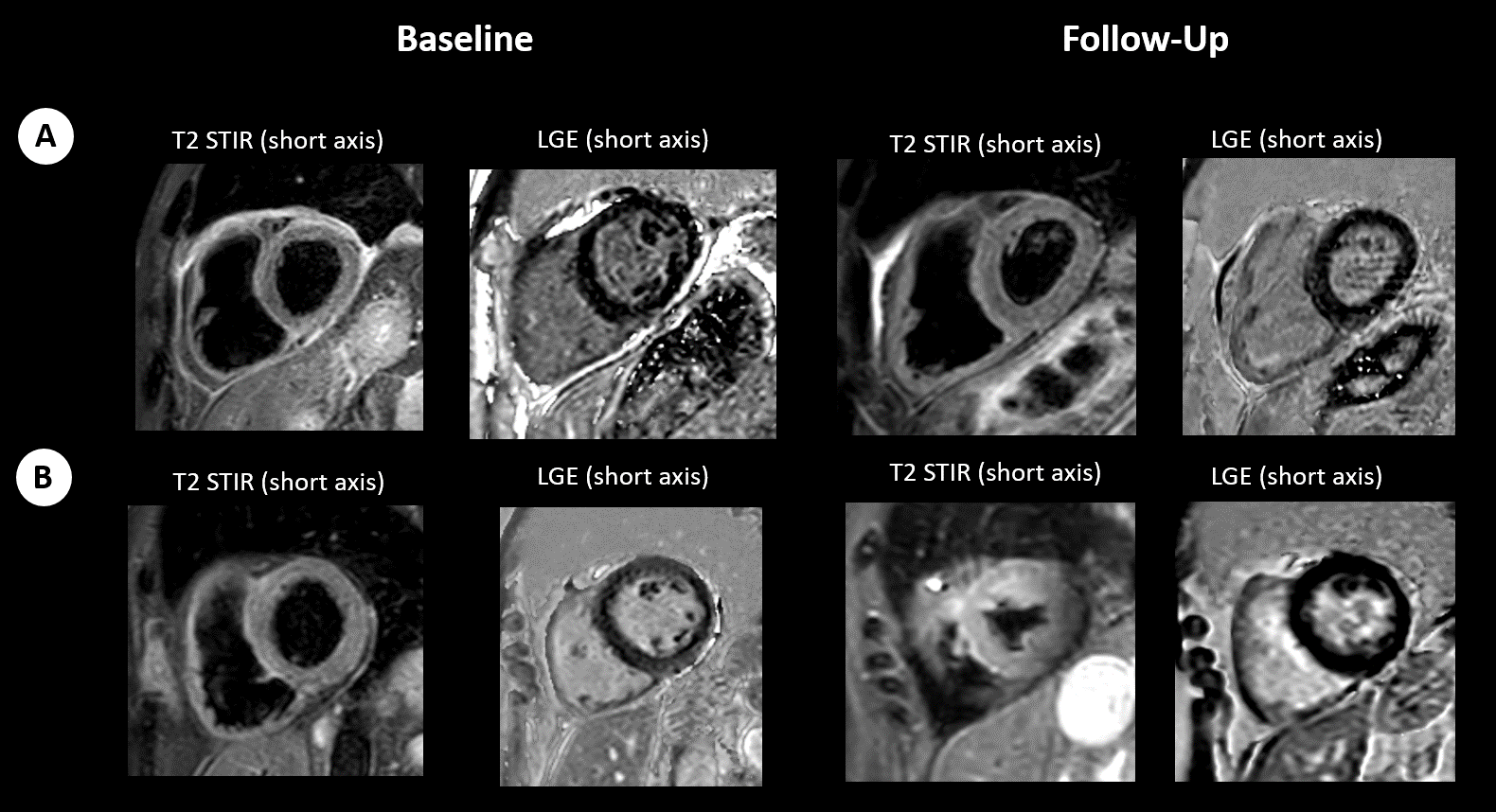

Supplement: Supplementary file 1 — Supplementary material [file mmc1.docx]
